# Telomeres are partly shielded from ultraviolet-induced damage and proficient for nucleotide excision repair of photoproducts

**DOI:** 10.1038/ncomms9214

**Published:** 2015-09-09

**Authors:** Dhvani Parikh, Elise Fouquerel, Connor T. Murphy, Hong Wang, Patricia L. Opresko

**Affiliations:** 1 Department of Environmental and Occupational Health, University of Pittsburgh, Pittsburgh, Pennsylvania 15213, USA; 2 University of Pittsburgh Cancer Institute, Pittsburgh, Pennsylvania 15213, USA; 3 Center for Nucleic Acids Science and Technology, Carnegie Mellon University, 4400 Fifth Avenue, Pittsburgh, Pennsylvania 15213, USA; 4 Department of Physics, North Carolina State University, Raleigh, NC 27695, USA

## Abstract

Ultraviolet light induces cyclobutane pyrimidine dimers (CPD) and pyrimidine(6–4)pyrimidone photoproducts, which interfere with DNA replication and transcription. Nucleotide excision repair (NER) removes these photoproducts, but whether NER functions at telomeres is unresolved. Here we use immunospot blotting to examine the efficiency of photoproduct formation and removal at telomeres purified from UVC irradiated cells at various recovery times. Telomeres exhibit approximately twofold fewer photoproducts compared with the bulk genome in cells, and telomere-binding protein TRF1 significantly reduces photoproduct formation in telomeric fragments *in vitro*. CPD removal from telomeres occurs 1.5-fold faster than the bulk genome, and is completed by 48 h. 6–4PP removal is rapidly completed by 6 h in both telomeres and the overall genome. A requirement for XPA protein indicates the mechanism of telomeric photoproduct removal is NER. These data provide new evidence that telomeres are partially protected from ultraviolet irradiation and that NER preserves telomere integrity.

## Abstract (web summary)

DNA damage caused by ultraviolet irradiation is removed from the genome by nucleotide excision repair; however, it is unclear if this occurs at chromosome ends. Here the authors provide evidence indicating that telomeres are partially shielded from damage and that repair is fully functional.

## Graphical abstract

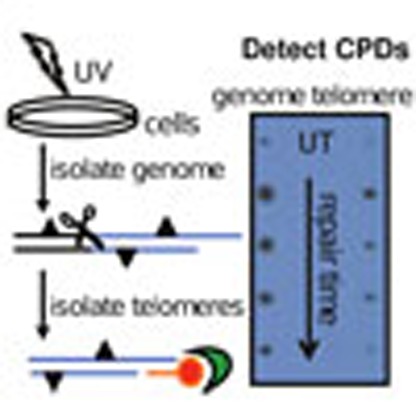

Genomic stability is essential for cellular health and survival. Since DNA damage from environmental and endogenous sources is inevitable, mechanisms for subsequent repair and restoration to undamaged DNA are required. Ultraviolet-light exposure generates DNA photoproducts in which two adjacent pyrimidines are covalently joined to form cyclobutane pyrimidine dimers (CPD) or pyrimidine(6–4)pyrimidone photoproducts (6–4PP)^1^. Unrepaired photoproducts are highly mutagenic and interfere with DNA replication and transcription^2^,^3^. Cellular mechanisms for managing photoproducts include global genome repair, transcription-coupled repair or translesion DNA synthesis^4^,^5^,^6^. Mammalian nucleotide excision repair (NER) accomplishes CPD and 6–4PP removal using an array of 30 different proteins^4^. Mutations in any one of seven NER proteins, including XPA protein, cause NER deficiency and the severe sunlight sensitivity and skin cancer-prone disorder xeroderma pigmentosum (XP)^7^. Global genome NER involves damage recognition and verification, dual-strand incisions flanking the lesion, repair synthesis and strand ligation^8^. Transcribed genes are repaired more rapidly than non-transcribed genes by the transcription-coupled NER pathway, which initiates when the RNA polymerase stalls at the lesion^6^. Finally, DNA polymerase η can accurately bypass CPDs during DNA replication to enable replication fork progression^9^. These mechanisms are essential for preserving the genome in the face of bulky lesions.

Both ultraviolet irradiation and telomere shortening are associated with skin aging and increased skin cancer risk^7^,^10^. Critically short or dysfunctional telomeres at chromosomal ends trigger cell growth arrest or apoptosis which drive aging-related pathologies, or chromosomal alterations, which drive carcinogenesis^11^,^12^. Telomeres in sunlight or UVB exposed skin tissue from humans and mice were shorter compared with unexposed skin^13^,^14^,^15^, suggesting a link between ultraviolet exposure and telomere alterations. Consistent with this, UVC irradiation induces telomere aberrations that are exacerbated in cells lacking polymerase η, indicating that unrepaired photoproducts can interfere with telomere replication^16^. Human telomeres at chromosome ends consist of about 1,500 tandem TTAGGG repeats, terminating with a 3′ single-stranded overhang that averages 100 nucleotides in length^17^,^18^. Previous studies demonstrate that telomeric repeats are susceptible to CPD formation following ultraviolet exposure^19^,^20^. The six-member shelterin protein complex at telomeres interacts with, and regulates, enzymes in every known DNA repair pathway including the NER endonuclease XPF-ERCC1 (refs 21, 22). Shelterin prevents inappropriate telomere processing by DNA repair enzymes, and inhibits homology directed repair and DNA double-strand break repair at telomeres^18^,^23^. Whether NER proteins function at damaged telomeres remains unresolved. Previous indirect approaches for lesion detection at telomeres led to equivocal results and were limited to CPD analysis in wild-type cell lines^19^,^20^.

Here we describe a novel direct approach to study NER at telomeres in which we isolate telomeres from UVC irradiated human cells and detect ultraviolet photoproducts using lesion specific antibodies and DNA blotting. Using this approach we show that both CPDs and 6–4PPs form at telomeres, but at levels lower than the bulk genome, and we provide evidence this may be due to shelterin protection. CPDs are removed from telomeres faster than from the bulk genome, while 6–4PPs are removed at similar rates. Furthermore, DNA photoproducts persist at telomeres from an XP-A patient, identifying NER as the mechanism for telomere photoproduct removal. Unrepaired photoproducts strongly inhibit shelterin TRF1 binding to telomeric DNA *in vitro*, underscoring the importance of NER at telomeres. To our knowledge, these studies provide the first evidence that NER is active at telomeres, and that NER restores telomeres that are damaged by ultraviolet light.

## Results

### Purification of telomeres from human cells

**Figure 1:**
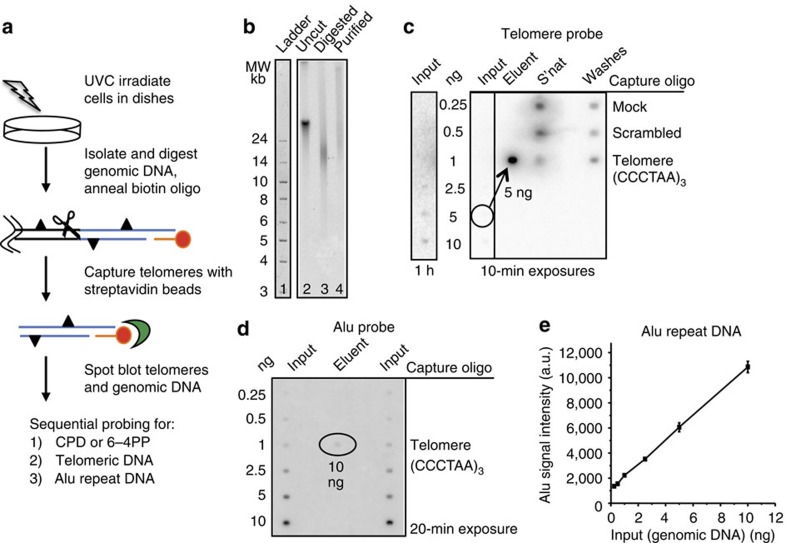
Telomere isolation assay. (**a**) Schematic of telomere capture assay. Telomeres (blue lines) are released by digesting the genome (scissors) and are captured by annealing a biotinylated oligonucleotide (red) that binds to the telomeric single-strand overhang and to streptavidin beads (green). Triangles denote photoproducts. (**b**) Undigested (lane 2) and digested (lane 3) genomic DNA, and isolated telomeres (lane 4) from BJ-hTERT cells were electrophoresed on a 0.6% agarose gel that was subsequently hybridized with a radiolabelled telomeric probe (lane 2–4). The ladder was visualized by SYBR Green staining (lane 1). (**c**) Specificity of telomere capture. Telomeres were isolated using three different conditions: mock (no oligonucleotide), scrambled (non-telomeric oligonucleotide) and telomere oligonucleotide (Table 1). Various amounts of digested genomic DNA (input), 50% of the unbound (s’nat) and 50% of the combined washes were loaded on the membrane. 50% of the eluent for the telomere oligo (exactly 5 ng) and total eluent for the mock (0 ng) and scrambled oligo (2.6 ng) was loaded. The membrane was hybridized with a radiolabelled telomeric probe and exposed to a phosphoimager screen for one hour or 10 min as indicated. (**d**) Telomere purity. Various amounts of digested genomic DNA (input) and exactly 10 ng of the telomere eluent were loaded on a membrane that was hybridized with a radiolabelled Alu repeat DNA probe. (**e**) Alu signal intensities from the genomic DNA were plotted against the DNA amounts loaded. Values and error bars represent the mean and s.d. from two independent experiments. The Alu signal intensity for 10 ng of telomere eluent corresponded to about 1.2 ng.

To study NER at telomeres we established an assay to directly measure photoproducts in telomeres isolated from ultraviolet-exposed cells. Since telomeres represent <0.026% of the human genome, we required large amounts of genomic DNA to obtain sufficient telomeres for analysis. We chose highly proliferative BJ skin fibroblasts engineered to express exogenous telomerase at an early passage before significant telomere shortening^24^. Telomeres were isolated from UVC irradiated and untreated human cells by annealing a biotinylated oligonucleotide that was complementary to the G-rich telomeric single-strand overhang^17^. Previous studies showed overhang lengths on leading and lagging strand telomeres are similar in BJ-hTERT cells, but can differ in primary cells^25^. The telomere/oligonucleotide complexes were captured with streptavidin-coated magnetic beads and washed, followed by the analysis of purified telomeres by spot blotting onto membranes (Fig. 1a). To obtain telomeric fragments, and to minimize the presence of sub-telomeric DNA, we digested the bulk genomic DNA with a cocktail of four frequent cutter restriction enzymes that do not recognize or cleave telomeric sequences^26^. Agarose gel resolution revealed that the bulk genome was completely digested to fragments of ≤1 kb (Supplementary Fig. 1). To examine the integrity of the purified telomeres we resolved the telomeric fragments obtained before purification (Fig. 1b, lane 3, digested) and after purification (lane 4, purified), on an 0.8% agarose followed by Southern blotting and hybridization with a radiolabelled telomere-specific probe. This analysis revealed that the average telomere length in our BJ-hTERT cell line is 17±1.1 kb (mean±s.d. from three independent experiments), and that the purified telomeres remained intact, although they migrated slightly slower than the unpurified telomere fragments (compare lanes 3 and 4, Fig. 1b). We suspect the elution of the telomeres at 50 °C may allow for some partial duplex melting and potential secondary structure formation that could retard the fragments during migration. This analysis indicates that any reduction in photoproducts observed in the repair experiments could not be attributed to telomere degradation during the isolation procedure.

To test the efficiency and specificity of telomere pulldown, we performed capture assays in the absence of biotinylated oligonucleotide (mock), with a biotinylated non-telomeric oligonucleotide (scrambled) or a biotinylated oligonucleotide containing 5′-(CCCTAA)_3_-3′ telomeric sequence (Table 1). From 100 μg of digested genomic DNA each, we recovered no detectable DNA for the mock control, 2.6 ng for the scrambled control, and 10 ng for the telomeric oligonucleotide (Fig. 1c). Supernatant, wash and eluents from the three capture experiments were loaded onto a membrane and hybridized with a radiolabelled telomeric probe. Exactly 5 ng of the eluent from the telomere capture oligonucleotide was loaded, and comparison with 5 ng of input DNA indicates a very strong enrichment for telomeres (Fig. 1c). The average efficiency of telomere purification was 33±0.06% (three independent experiments), calculated as [bound÷(bound+unbound)], and agreed with previous reports^17^. This value closely matches the recovery efficiency of 38% calculated by measuring DNA yields (see Methods for calculation). The actual telomere yield of 10±1.3 ng (average from five independent experiments) was divided by the maximal telomere yield (26 ng) that could be obtained from 100 μg of genomic BJ-hTERT DNA. Similar telomere yields of 10±3 ng were obtained from cells following UVC irradiation at 10 J m^−2^, indicating that ultraviolet photoproduct formation did not alter the efficiency of telomere isolation.

To estimate the purity of the eluted telomeres we loaded various amounts of input (genomic DNA) and 10 ng of purified telomere eluent, followed by hybridization with a radiolabelled probe against Alu repeat DNA (Fig. 1d). Alu repeats are short interspersed elements that comprise ∼10% of the genome and are commonly used as a negative control for identifying telomere-binding proteins in chromatin immunoprecipitation assays^27^,^28^. Based on signal intensities quantified for genomic DNA, the total amount of non-telomeric DNA present in the telomere eluent is ∼1.2±0.2 ng (mean±s.d. from two independent experiments; Fig. 1e). This indicates that at least 90% of the DNA present in the eluent is enriched telomeres.

### Telomeres show formation and removal of CPDs and 6–4PPs

**Figure 2:**
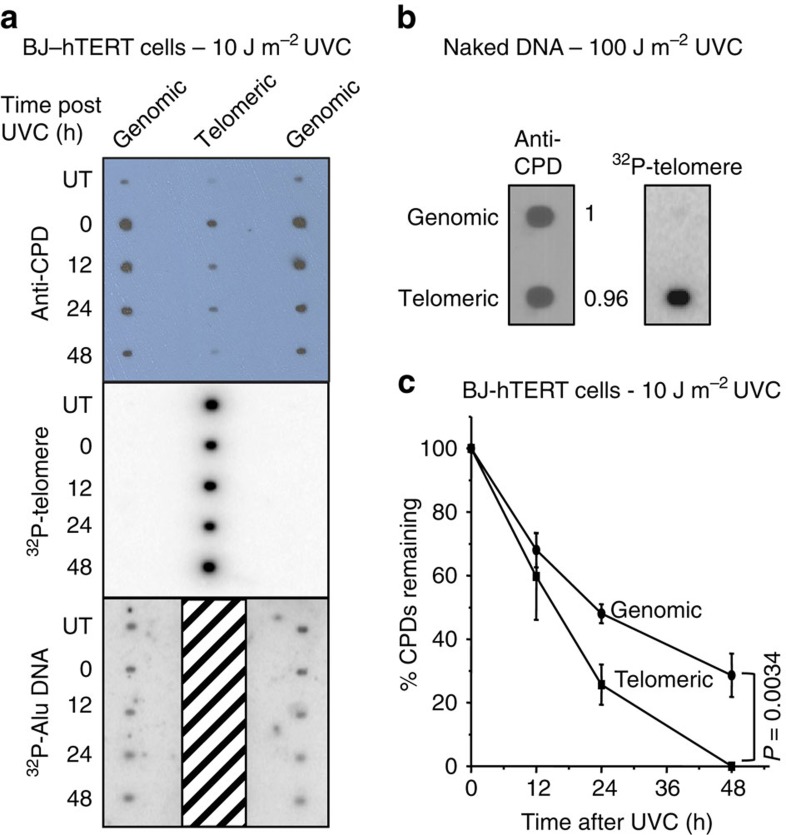
Quantification of CPD formation and removal in telomeres from UVC exposed BJ-hTERT. (**a**) Cells were untreated or exposed to 10 J m^−2^ UVC followed by harvesting at various repair times (0–48 h). Telomeres were isolated from purified genomic DNA (100 μg each time point) and loaded on blots (7 ng, lane 2) with equal amounts of genomic DNA (7 ng, loaded in duplicate lanes 1 and 3). The blot was sequentially probed with a CPD antibody, a radiolabelled telomere probe, and a radiolabelled Alu repeat probe. The telomere probes remained bound (hashed box) since membranes could not be stripped without losing DNA. (**b**) Purified genomic DNA (100 μg) was exposed to 100 J m^−2^ UVC *in vitro*. Telomeres were isolated and blotted (3 ng) with equal amounts of genomic DNA, and probed with a CPD antibody and radiolabelled telomere probe. The CPD signal intensity was quantitated and normalized to genomic DNA for comparison; from three independent experiments. (**c**) The CPD signal intensity was quantitated for experiments in **a**, normalized to 0 h, and plotted against recovery time. Values and error bars are the mean and s.e.m. from three independent experiments. The difference between the curves is statistically significant (*P*=0.0034) by two-way ANOVA.

Photoproduct removal in genomic and telomeric DNA of NER proficient BJ-hTERT cells exposed to 10 J m^−2^ UVC was quantified at various recovery times by immunospot blot assay. This exposure generated ∼3.6 CPDs per 10 kb of genomic DNA (Supplementary Fig. 2), in general agreement with previous reports^29^,^30^. This value was derived by comparing signal intensities from CPD immunodetection in genomic DNA with those obtained from lambda DNA standards for which the ultraviolet lesion frequencies were determined by quantitative PCR (qPCR)^31^ (Supplementary Fig. 2). We confirmed previous reports that CPDs form in telomeric DNA^19^,^20^. However, the CPD signal for equal amounts (7 ng) of loaded telomeric DNA at 0 h recovery was on average 2.6-fold (±0.32 s.e.m.) lower compared with that for bulk genomic DNA (Fig. 2a, top panel). This equates to about 1.4 CPD per 10 kb of telomeric DNA, or 2.3 CPDs per 17 kb telomere. In contrast, naked telomeres isolated from purified genomic DNA that was UVC exposed (100 J m^−2^) *in vitro*, displayed a similar CPD signal intensity (0.96±0.5 s.e.m.) relative to genomic DNA (Fig. 2b).

Following cellular 10 J m^−2^ UVC exposure, the CPD signal decreased progressively with increasing recovery time for both bulk genomic and telomeric DNA, however, the reduction was 1.5-fold more rapid for telomeric DNA (Fig. 2c). The rate difference was based on the slopes calculated from the linear portions of the curves (0 to 24 h). The difference between the curves for telomeric versus genomic CPD repair is statistically significant (*P*=0.0034, two-way ANOVA). Subsequent hybridization with the radiolabelled telomeric probe confirmed successful enrichment of telomeric DNA in the purified samples and equal loading of telomeric DNA for each recovery time point (Fig. 2a). Membrane stripping led to telomere loss, therefore, membranes were subsequently hybridized with the radiolabelled Alu repeat DNA probe to confirm equal loading of the genomic DNA samples (Fig. 2a).

To ensure that the reduction in CPDs was not due to dilution through cell division, we examined cell proliferation by obtaining cell counts for each repair time point after the 10 J m^−2^ UVC exposure. UVC irradiation normally triggers transient cell cycle arrest^32^. Untreated BJ-hTERT cells doubled in number by 48 h, while the UVC exposed cells failed to double even after 72 h recovery (Supplementary Fig. 3a). Since telomerase is active in S-phase^33^,^34^, telomere elongation should not occur during the cell cycle arrest. Regardless, cells that over express telomerase exhibit lengthening by 60 to 120 nucleotides per telomere per cell cycle^35^, which represents a minor fraction of total telomeric DNA in BJ-hTERT cells (0.35–0.70% based on 17-kb length). Furthermore, about 95% of the cells collected via trypsinization for the repair assay were alive as determined by trypan blue staining (Supplementary Fig. 3b). These results confirm that repair assays were conducted on viable cells, and that the observed reductions in photoproducts were not due to cell division.

**Figure 3:**
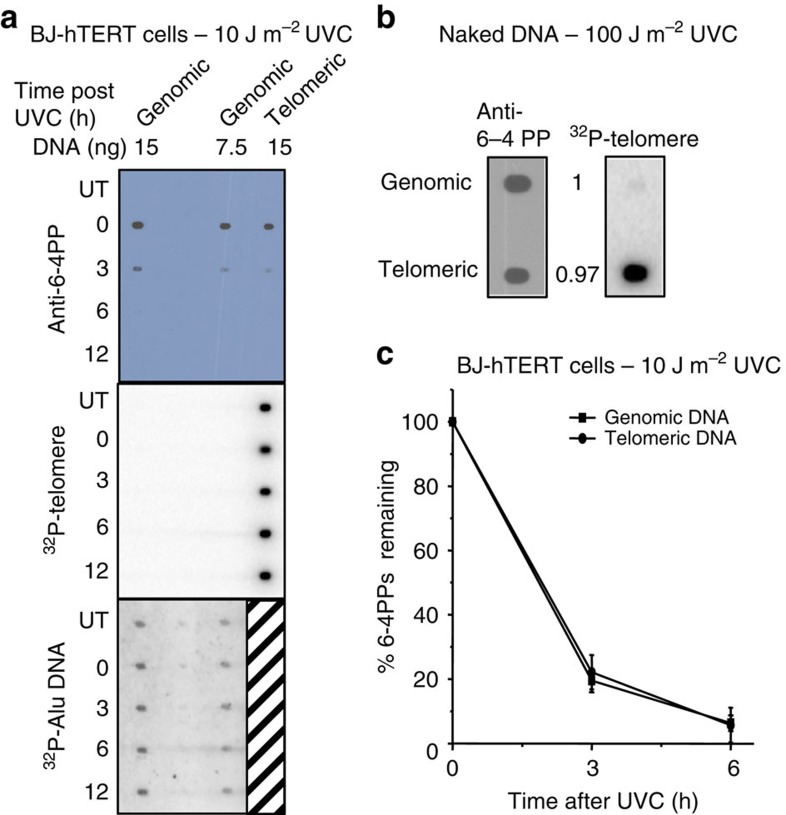
Quantification of 6–4PP formation and removal in telomeres from UVC exposed BJ-hTERT cells. (**a**) Cells were untreated or exposed to 10 J m^−2^ UVC followed by collecting at various repair times (0–12 h). Telomeres were isolated from purified genomic DNA (100 μg each time point) and combined from two separate experiments to obtain 15 ng (lane 3) for loading. Genomic DNA was loaded at 15 ng (lane 1) and 7.5 ng (lane 2). The blot was sequentially probed with a 6–4PP antibody, a radiolabelled telomere probe, and a radiolabelled Alu repeat probe. (**b**) Purified genomic DNA (100 μg) was exposed to 100 J m^−2^ UVC *in vitro*. Telomeres were isolated and blotted (7 ng) with equal amounts of genomic DNA and probed with the 6–4PP antibody and radiolabeled telomere probe. The 6–4PP signal intensity was quantitated and normalized to genomic DNA for comparison; from three independent experiments. (**c**) The 6–4PP signal intensity was quantitated for experiments in **a**, normalized to 0 h, and plotted against recovery time. Values and error bars are the mean and s.e.m. from four independent experiments for genomic DNA and two experiments for telomeric DNA. The difference between the curves was not statistically significant (*P*=0.95) by two-way ANOVA.

The other common photoproduct 6–4PP had not been previously examined at telomeres. These lesions induce greater distortion in the duplex DNA and are repaired more rapidly than CPDs, however, they are formed at a lower frequency^36^,^37^. The 10 J m^−2^ UVC exposure generated about 1.4 6–4PPs per 10 kb of genomic DNA (Supplementary Fig. 2). Therefore, higher amounts of loaded purified telomeres (15 ng isolated from 200 μg genomic DNA) were required for reliable 6–4PP detection. Following 10 J m^−2^ UVC, the 6–4PP signal was on average 1.9-fold (±0.32 s.e.m.) lower for isolated telomeric DNA, compared with equal amounts (15 ng) of bulk genomic DNA (Fig. 3a). This equates to ∼0.74 6–4PPs per 10-kb telomeric DNA, or 1.2 6–4PPs per 17-kb telomere. As for CPDs, we observed similar amounts of 6–4PP formation on *in vitro* UVC irradiation (100 J m^−2^) of naked telomeric DNA (0.97±0.15 s.e.m.) relative to naked genomic DNA (Fig. 2b). This suggests that telomeres in cells may be partly protected from ultraviolet irradiation.

During recovery from cellular irradiation, the 6–4PPs were removed at similar rates in bulk genomic DNA compared with telomeric DNA, and were removed more rapidly than CPDs (Fig. 3c). About 20% of the 6–4PPs remained in both genomic and telomeric DNA by 3 h and only ∼6% remained by 6 h post-UVC exposure. Hybridization with telomeric and Alu-repeat-specific probes confirmed equal loading of telomeric DNA and genomic DNA, respectively, for all time points (Fig. 3a). Finally, 6–4PP removal from telomeric DNA was not dependent on telomerase. We observed nearly complete removal of 6–4PP in both bulk genomic and isolated telomeric DNA by 12 h post UVC in the telomerase negative human osteosarcoma cell line U2OS (Supplementary Fig. 4). The initial amount of 6–4PPs formed in telomeric DNA from U2OS cells was about twofold lower compared with bulk genomic DNA, similar to BJ-hTERT cells (Supplementary Fig. 4). Repair rates of both 6–4PP and CPDs in genomic DNA were slower in U2OS cells compared to BJ-hTERT (Supplementary Figs 4 and 5). U2OS cells use the alternative lengthening of telomeres (ALT) pathway, and ALT cells contain extrachromosomal telomere-repeat (ECTR) DNA. However, ECTR comprises <4% of the telomeric repeat DNA in U2OS cells^38^. Of the ECTR species, G-circles consisting of single-stranded TTAGGG repeats could potentially anneal with the telomere capture oligonucleotide. Therefore, we confirmed that the isolated telomere fractions lack detectable G-circles (Supplementary Fig. 4c), thereby, validating photoproduct detection in U2OS telomeres. In summary, our data demonstrate that UVC exposure induces 6–4PPs at telomeres, although at levels lower than the bulk genome, and that 6–4PPs are rapidly removed from both telomeres and the bulk genome in a telomerase-independent manner.

### TRF1 protects telomeric DNA from photoproduct formation

**Figure 4:**
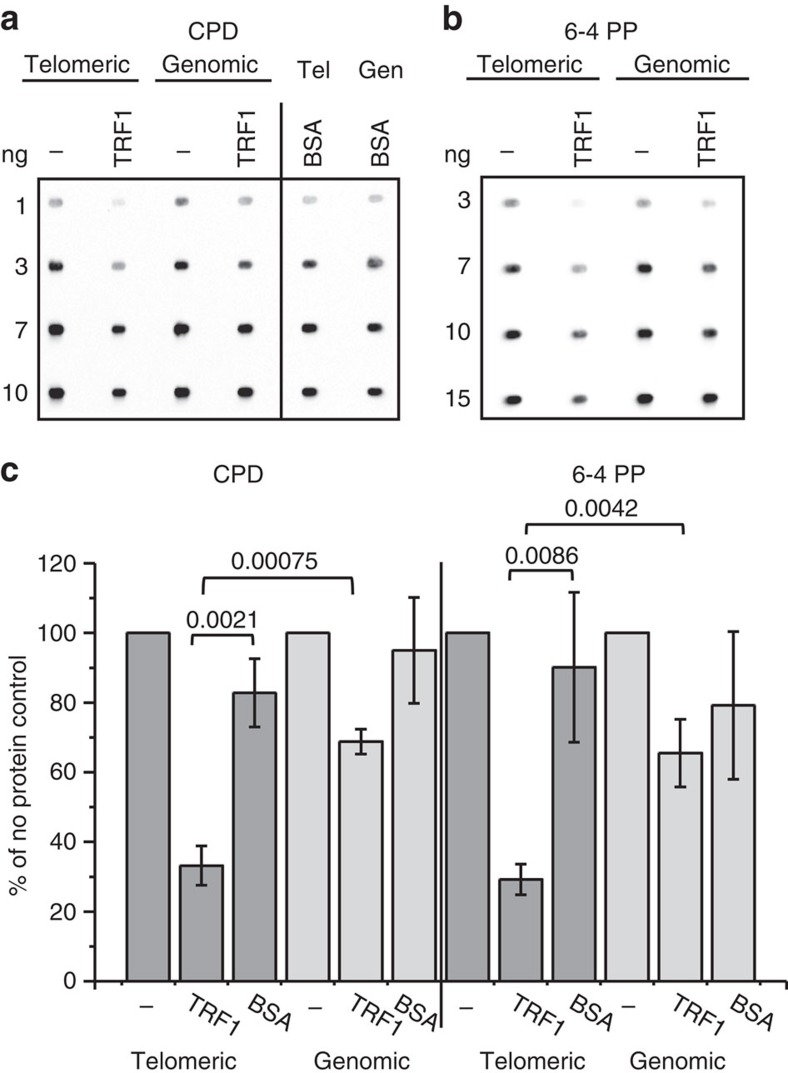
TRF1 suppresses UVC-induced CPD and 6–4PPs formation in telomeric fragments. (**a**) Purified duplex DNA restriction fragments (25 μg ml^−1^) containing (TTAGGG)_270_ repeats (1.6 kb, telomeric) or non-telomeric sequence (1.5 kb, genomic) were exposed to 100 J m^−2^ UVC. Before irradiation fragments were preincubated for 45 min at room temperature with either no protein (−), 2 μM TRF1, or 2 μM BSA. Various amounts of fragments from each reaction were immunoblotted for CPDs (**a**) or 6–4PPs (**b**). The antibody signal intensities were quantitated, and amounts within the linear range (3 ng for CPD and 7 ng for 6–4PP) were normalized to the no protein controls, and plotted for each condition. Values and error bars are the mean and s.d. from three independent experiments. The differences between telomeric DNA+TRF1 versus telomeric DNA+BSA and telomeric DNA+TRF1 versus genomic DNA+TRF1 were statistically significant by one-way ANOVA; *P*-values indicated in the figure.

We predicted the mechanism for reduced photoproduct formation at telomeres *in vivo*, but not *in vitro*, may be shelterin binding, based on evidence that transcripion factors can inhibit ultraviolet-induced photoproduct formation at promoters when bound^39^. To test this we isolated a 1.6-kb restriction fragment from plasmid pSXneo 270(T2AG3) containing 270 TTAGGG repeats (telomeric) and a 1.5-kb fragment containing non-telomeric sequence (genomic)^40^. The frequency of dipyrimidine sequences is only slightly higher in telomeric (67%) versus genomic (53%) fragments (Supplementary Table 1). Duplex fragments (25 nM) were preincubated in buffer alone or with 2 μM purified shelterin protein TRF1 or bovine serum albumin (BSA). Since TRF1 binds to 2.5 telomeric repeats as homodimers^41^, this represents a 1:2.7 ratio of TRF1 dimer to binding sites, or about 25 dimers per kb DNA. Previous work estimated about 5 to 20 TRF1 dimers per kb telomeric DNA depending on the cell line^42^. Fragments were then irradiated with 100 J m^−2^ UVC and immunoblotted for CPDs and 6–4PPs (Fig. 4a,b). For naked DNA fragments the ratio of CPD and 6–4PP formed in non-telomeric DNA versus telomeric DNA was 0.96±0.11 and 1.1±0.16, respectively, indicating no obvious bias for photoproduct formation in telomeric repeats. TRF1 preincubation reduced CPD and 6–4PP formation to 33 and 29% relative to naked telomeric DNA, respectively (Fig. 4c). In contrast TRF1 provided less protection to non-telomeric DNA causing a modest reduction in CPDs and 6–4PPs to 69 and 66% relative to naked DNA, respectively. Preincubation with BSA indicated that protein in solution was able to impart some shielding of the DNA from UVC irradiation, but TRF1 protection of telomeric DNA was significantly greater than BSA (Fig. 4c). Our data demonstrate that telomere-binding proteins shield telomeric DNA *in vitro*, and provide evidence that shelterin may contribute to lower photoproduct formation at telomeres compared with the bulk genome in cells.

### Removal of 6–4PPs at telomeres depends on XPA protein

**Figure 5:**
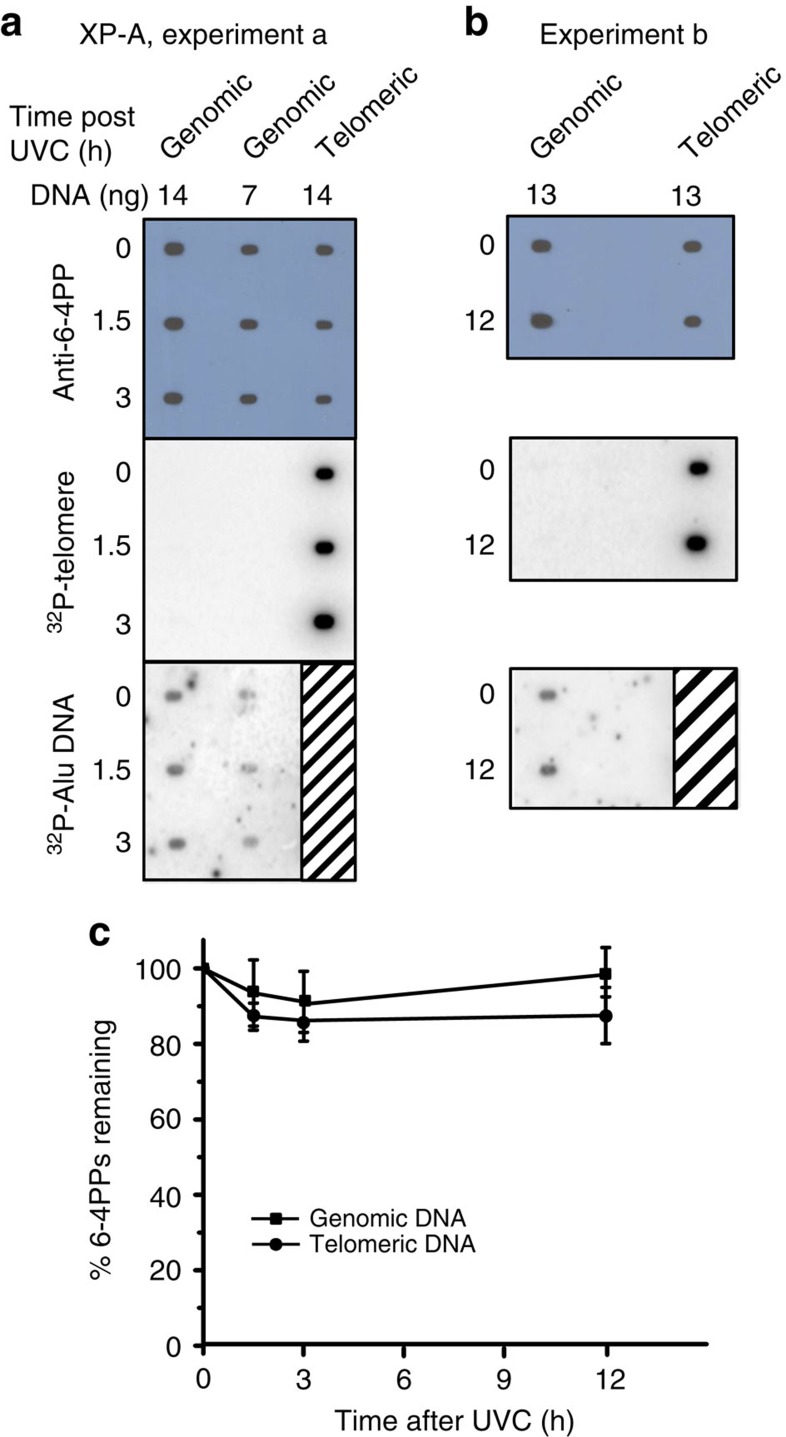
XPA protein is required for 6–4PP removal from telomeres. (**a**,**b**) Cells were exposed to 10 J m^−2^ UVC and harvested at various repair times (0, 1.5 and 3 h, experiment **a**; 0 and 12 h, experiment **b**). Telomeres were isolated from purified genomic DNA (200 μg each time point) to obtain 13–14 ng (lane 3, **a**; lane 2, **b**) for loading. Genomic DNA was loaded at amounts indicated. The blots were sequentially probed with a 6–4PP antibody, a radiolabelled telomere probe, and a radiolabelled Alu repeat probe. (**c**) The 6–4PP signal intensity was quantitated, normalized to 0 h, and plotted against recovery time. Genomic DNA values represent the mean and s.e.m. from four independent experiments, and values and error bars for telomeres were derived from two independent experiments for each time point. The difference between the curves was not statistically significant (*P*=0.17) by two-way ANOVA.

To determine whether the mechanism of photoproduct removal at telomeres is by NER, we repeated the telomere capture and immunoslot blot assays in cells lacking repair. NER deficent skin fibroblasts were obtained from an XP-A individual and were immortalized by SV40 transformation^43^. We first confirmed that the XP-A cells were more sensitive to UVC compared with repair proficient BJ-hTERT cells by conducting a long-term survival assay^44^. For this experiment cells were UVC exposed and allowed to recover for 6 h, then sub-cultured for 7 days and counted (Supplementary Fig. 3c). Although sensitive to UVC, the XP-A cells collected shortly after UVC irradiation for the repair assays were at least 95% viable as determined by trypan blue staining (Supplementary Fig. 3b). Genomic DNA isolated from XP-A cells lacked 6–4PP and CPD repair up to three or 48 h, respectively, post UVC exposure (Fig. 5a and Supplementary Fig. 6). Similarly, there was no significant change in the amount of 6–4PPs in the isolated telomeres by 3 h, compared with 0 h, post UVC exposure (Fig. 5a,c). Even when the recovery time was extended to 12 h, we still did not observe a significant reduction in 6–4PPs in either bulk genomic or telomeric DNA (Fig. 5b,c). These results validate our approach for the ability to detect a lack of repair in telomeres, and provide evidence that NER is active at telomeres and removes photoproducts.

### An unrepaired CPD inhibits TRF1 binding

**Figure 6:**
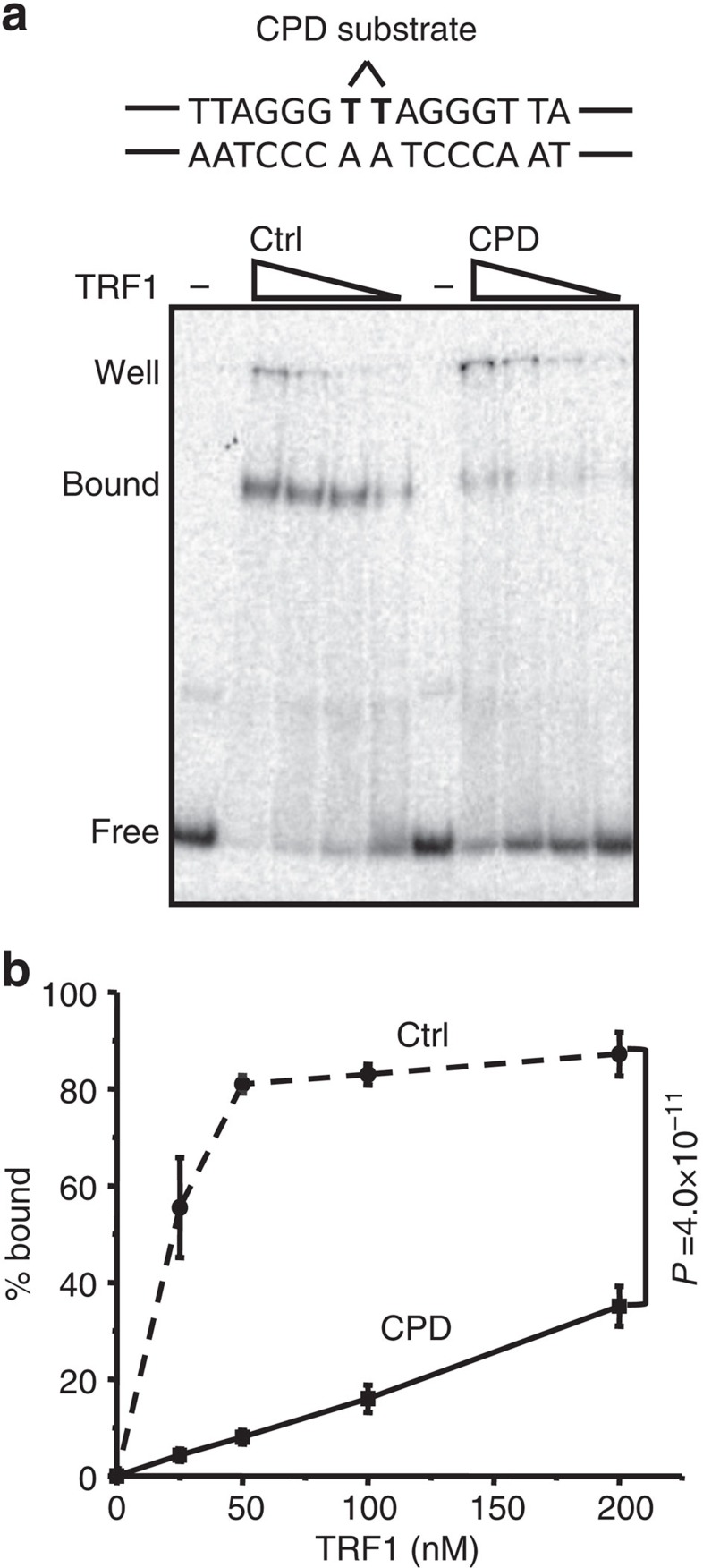
A cyclobutane pyrimidine dimer inhibits TRF1 binding to telomeric DNA. The TRF1 homodimer binding sequence and site specific CPD (⁁) is shown. (**a**) Substrates (2.5 nM) consisting of annealed Ctrl/TPL or CPD/TPL duplexes were incubated with decreasing TRF1 concentrations (200, 100, 50 or 25 nM) for 20 min in binding buffer, and reactions were run on a 5% acrylamide native gel. Bound and unbound (free) substrate are indicated. (**b**) The per cent bound was calculated and plotted against TRF1 concentration. Circle and dotted line, control (Ctrl); square and solid line CPD-containing substrate (CPD). Values and error bars indicate the mean and s.d. from three independent experiments. The difference between the curves was statistically significant; *P*=4.0 × 10^−11^ by two-way analysis of variance.

We showed that photoproducts persist in cells lacking NER (Fig. 5). Telomeres could potentially tolerate an accumulation of unrepaired DNA lesions if such lesions fail to disrupt shelterin binding. However, previous reports show that 8-oxo-guanine decreases TRF1 and TRF2 binding to telomeric duplexes by 50% *in vitro*^45^. To determine whether unrepaired photoproducts also inhibit TRF1 binding, we performed gel shift assays with purified TRF1 and a 39 bp duplex DNA substrate containing the minimal consensus binding sequence for a TRF1 homodimer^45^. A damaged substrate was constructed by replacing the central adjacent thymine with a CPD lesion (Table 1). We tested a CPD since it distorts the helix less than a 6–4PP^6^ and is commercially available. Migration of telomeric substrates through the agarose gel was retarded due to TRF1 binding to the duplex (Fig. 6a and Supplementary Fig. 7). The presence of a single CPD lesion within the telomeric substrate caused a prominent decrease in TRF1 binding to the substrate (lanes 7 to 10); 14-fold (93%) decrease within the linear range of the binding curve (25 nM TRF1) compared with the control (Fig. 6b). These data suggest that persistent CPDs at the telomeres could alter shelterin binding, underscoring the potential importance of repair at telomeres.

## Discussion

In this study we established an assay for directly visualizing and quantifying photoproduct formation and removal in telomeres isolated from various UVC irradiated cell lines. Using this approach we confirmed CPD formation in telomeres, and discovered that telomeres are also susceptible to 6–4PP formation (Figs 2 and 3). The frequency of photoproducts at telomeres was about twofold lower compared with the bulk genome *in vivo*, and our results with purified components *in vitro* provide evidence that shelterin binding may partly shield the telomeres from damage. Using the telomere isolation and immunoblotting approach we observed that CPDs and 6–PPs are removed from telomeres, and that lesion reduction requires the NER protein XPA, but does not depend on telomerase activity. We discovered that a single unrepaired CPD strongly inhibited shelterin TRF1 binding to telomeric DNA *in vitro*, suggesting that unrepaired lesions at telomeres could be deleterious if they accumulate. To our knowledge this study provides the first direct evidence that NER functions at telomeres, and is essential for photoproduct removal to restore damage telomeres.

Our result that CPDs and 6–4PPs are less abundant at telomeres compared with the bulk genome suggests that shelterin may partly protect the telomeres from ultraviolet irradiation. *In vitro* irradiation of naked genomic DNA and telomeres (Figs 2b and 3b), and of purified 1.5-kb duplex fragments from plasmids (Fig. 4), indicate that telomeric repeats are not significanlty less (or more) susceptible to photoproduct formation than non-telomeric sequences. Our results differ from studies that showed telomeric oligonucleotides are more susceptible to UVC-induced CPD formation *in vitro*, compared with non-telomeric oligonucleotides^19^, and may reflect differences in oligonucleotides compared with restriction fragments and isolated telomeres. The bulk of the telomeres are duplex repeats. Our results show purified telomere binding protein TRF1 suppresses damage in telomeric duplex DNA *in vitro* (Fig. 4), similar to some transcripion factors. which can inhibit photoproduct formation at bound promoters^39^. Shelterin consists of six proteins, including TRF1 and TRF2, which bind duplex telomeric DNA, and POT1 which binds single-stranded telomeric DNA^18^. Furthermore, TRF2 causes compaction and TRF1 leads to looping of telomeric DNA^41^,^46^, which may influence efficiency of photoproduct formation. Therefore, it is reasonable to predict that the full shelterin protein complex likely provides greater protection at telomeres, than the single TRF1 factor tested here. However, while our studies revealed telomeres are less susceptible to photoproduct formation compared with the bulk genome, we cannot rule out the possibility that telomeres may be more sensitive than specific sites within the genome. Previous work reported more UVC-induced CPDs at telomeric fragments compared with fragments from the *p53* or 28S rDNA genes^19^. In summary, our data provide evidence that the shelterin complex at telomeres modulates susceptibility to photoproduct formation.

Global genome repair (GGR) removes photoproducts and bulky lesions from both transcribed and silent genomic regions, whereas transcription-coupled repair (TCR) is a specialized mechanism limited to lesion removal on the template DNA strands of actively transcribed genes^6^. Therefore, our analysis of photoproduct removal from the bulk genome represents primarily GGR rates and is consistent with CPD and 6–4PP rates reported elsewhere for human cells^19^,^20^,^47^. However, telomeres are transcribed from the C-rich strand into non-coding RNAs called TERRA which are required for telomere homeostasis^48^,^49^. Therefore, photoproduct removal rates from telomeres may include TCR of CPDs at CT or CC dipyrimidines on the actively transcribed telomeric strand. This may explain why we observed a 1.5-fold faster removal of CPDs from isolated telomeres compared to bulk genomic DNA (Fig. 2). TCR of 6–4PPs is often obscured because these lesions are rapidly and efficiently repaired by GGR^6^,^37^,^50^, in agreement with our result that 6–4PP removal rates are similar in telomeres compared to bulk genomic DNA (Fig. 3).

Using our telomere capture and immunospot blot approach we observed that only 25% of the detectable CPDs remain at telomeres by 24 h of repair, and that they disappear by 48 h, in BJ-hTERT fibroblasts (Fig. 2). This rate is higher than that reported previously for telomere CPD removal in primary fibroblasts derived from normal young individuals, in which 50% of the telomeric CPDs remained by 24 h similar to repair rates in non-transcribed strands^20^. However, direct comparison is difficult since the ultraviolet dose used was higher (20 J m^−2^) than in our experiments, and the primary fibroblasts lacked telomerase and possessed shorter telomeres; all factors that may influence telomere repair rates. For example, if efficient telomere repair relies on transcription or TCR, perhaps differences in the levels of telomere transcription among cell lines may influence the efficiency of telomere repair. Importantly, both our approach and this previous approach^20^ yielded the similar result that ultraviolet-induced CPDs are removed from telomeres during recovery. These approaches have in common the detection of CPDs in telomere restriction fragments either by antibody staining of isolated telomeres (this study) or by T4 endonuclease cleavage of unpurified telomere fragments^20^.

Telomeres released by restriction enzymes retain some sub-telomeric DNA that may be resistant to digestion. We attempted to minimize sub-telomeric DNA by using multiple restriction enzymes that are known to cut within the sub-telomeric regions^26^. This region contains degenerate telomeric repeats and is estimated to average ∼3.5 kb in BJ-hTERT cells^51^. Therefore, ∼20% of the purified telomere fragments could contain sub-telomeric DNA. We cannot rule out the possibility that some of the CPD and 6–4PP removal in the purified telomeres is due to repair in sub-telomeric regions. However, if the telomeres were resistant to repair then photoproducts should have remained visible since the majority of the isolated telomeres consists of telomeric DNA (Figs 2, 3 and 5). In constrast, we failed to detect CPDs after 48 h and 6–4PPs after 6 h of repair in telomeres from BJ-hTERT cells (Figs 2 and 3). Another possibility is that telomeres are refractory to CPD and 6–4PP formation and that lesions are limited to the sub-telomeric region. This is highly unlikley due to the small size of the target; 10 J m^−2^ induces at least one CPD per 10 kb and 6–4PPs are about three-fold less frequent^52^,^53^. Furthermore, a previous approach reported CPDs in telomeric DNA by using quantitative PCR to measure telomeric DNA, which excludes sub-telomeric DNA^19^.

Disparity between our results and a previous study that reported no significant CPD removal from telomeres by 48 h^19^, may be explained partly by differences in cell lines or approaches. In the previous study denatured ssDNA from UV irradiated cells was chromatin immuno-precipitated with a CPD-antibody to isolate ssDNA fragments containing CPDs, followed by gene and telomeric DNA identification using quantitative PCR^19^. We used the reverse approach in which we isolated telomeres first, and then blotted them on a membrane for lesion detection with the CPD or 6–4PP antibodies. Telomeres present a challenge for lesion detection because the DNA must be denatured for the antibodies to recognize the lesion, and single-stranded telomeric G-rich repeats can fold into G-quadruplex structures^54^. Using our approach the telomeres are denatured in a membrane and fixed to avoid secondary structure formation, therefore, we do not suspect that G-quadruplex formation could prevent lesion detection. Furthermore, our approach is able to detect 6–4PPs in telomeres at all time points in NER-deficient cells (Fig. 5). Perhaps, a subset of telomeres retain photoproducts but are beyond the detection limit of our assay, and may be detectable by the more sensitive qPCR approach^19^. Another difference is that we used skin fibroblasts that are telomerase positive and an osteosarcoma cell line that maintains telomeres by ALT, rather than primary fibroblasts. A limitation of our assay is that it requires a large amount of genomic material and thus, highly proliferative cells. However, telomerase does not enable 6–4PP removal from telomeres (Supplementary Fig. 4) and previous studies also showed CPD removal from telomeres occurs in primary fibroblasts^20^. Another factor may be related to differences in transcriptional activity levels at telomeres among cell lines, which would influence lesion removal at telomeres by TCR as mentioned earlier.

The finding that 6–4PPs persist at telomeres in NER deficient cells, but are removed in NER proficient cells, provides strong evidence that NER is active at telomeres. Previous studies of CPD formation and removal at telomeres in normal cells could not rule out the possibility that lesion removal was accomplished by another mechanism^20^. Shelterin protein TRF2 prevents inappropriate cleavage of the telomeric 3’ ssDNA overhang by the key NER nuclease XPF-ERCC1 (ref. 21). Our data indicates this TRF2 inhibitory effect does not extend to NER. Thus, both NER (this study) and base excision repair are active at telomeres^55^, while homologous recombination repair and non-homologous end joining are suppressed at telomeres^18^,^23^. Together with reports that UV exposure apparently alters telomere lengths in tissue^13^,^14^,^15^, our finding provides evidence that repair of photoproducts is likely important for telomere preservation. Furthermore, we observed that an unrepaired CPD inhbited TRF1 binding to telomeric DNA by 14-fold, which is much greater than the 2-fold inhibition caused by an 8-oxo-guanine lesion on the identical substrate^45^. This strongly suggests that lack of lesion repair at telomeres could be deleterious if unrepaired lesions increase in density overtime. Our discovery that NER is active at telomeres is consistent with the prediction that UV photoproducts disrupt telomere function and therefore, need to be removed.

## Methods

### Cell culture and exposures

Telomerase immortalized human foreskin fibroblasts BJ-hTERT cells and human osteosarcoma U2OS cells were obtained from ATCC. The SV40-immortalized NER deficient human skin fibroblasts (GM04312) derived from an XP-A donor (XP20S) was obtained from the Coriell Cell Repository. This cell line harbours homozygous inactivating mutations in gene encoding XPA protein. Cells were grown at 37 °C and 5% CO_2_ in DMEM complete media containing 10% fetal bovine serum, penicillin (50 units ml^−1^) and streptomycin (50 μg ml^−1^) (Life Technologies). UVC irradiation was performed via a 254 nm wavelength emitting germicidal lamp on cells at 80% confluency in dishes lacking media. UVC exposures were measured with a UVX31 meter (UVP, Upland, CA). After exposures cells were incubated in fresh media, then washed with PBS and harvested at various repair time points.

### Cell viability and proliferation assays

For short term proliferation assays the cells were UVC irradiated (10 J m^−2^) or not in 60 mm dishes, incubated in fresh media, and then counted in duplicate at various repair time points (0 to 72 h) using a Beckman Coulter Z1 Cell Counter. The average cell number for each repair time point was divided by the cell number at 0 h recovery. Cell viability was determined by trypan blue exclusion. Per cent viability was calculated as [1.00—(number of blue cells÷number of total cells)] × 100. For long-term cell viability assays cells were irradiated with UVC (0, 5 or 10 J m^−2^) or not (untreated) and incubated in fresh media. After 6 h of recovery the cells were collected by trypsinization and counted, and then sub-cultured by seeding equal numbers of cells per 10-cm culture dish in duplicate. Following a seven day subculture, the cells were then counted.

### Genomic DNA and telomere purification

Genomic DNA was isolated from harvested cells using the Qiagen 20/G or 100/G DNA isolation kit. Approximately 20 × 10^6^ cells were harvested from eleven 100 mm dishes to yield about 100 μg of bulk genomic DNA per repair time point. Telomere isolation was based on a published method with some modfication^17^. Double stranded genomic DNA (100 μg) was digested overnight with AluI, HinfI, HphI and MnlI (0.5 U μg^−1^) restriction enzymes in 250 μl reaction volume to release intact telomeric fragments. Reactions were adjusted to 1 × SCC and 0.1% Triton X-100, and the digested DNA was then annealed with a biotinylated oligonucleotide (3.5 pmol) by controlled stepwise cooling from 80 to 25 °C (1.2 °C min^−1^) using a thermocycler. Then streptavidin-coated magnetic beads (18 μl, Invitrogen, M-280) prewashed with 1 × PBST and blocked with 5 × Denhardt solution, were incubated with the annealed samples overnight in a rotator end-over-end at 6 r.p.m. and 4 °C. Beads were collected against the side of the tubes by applying a magnet (Invitrogen), and unbound supernatants and subsequent washes were collected. The beads were washed three times with 1 × sodium chloride–sodium citrate (SSC), 0.1% Triton X-100, twice with 0.2x SSC and once with elution buffer (1 mM Tris pH 7.5, 1 mM EDTA, 10 mM LiCl). Beads were resuspended in 30 μl elution buffer and telomeres were slowly eluted by heating the tubes at 50 °C for 20 min. The elution was repeated with 15 μl. Telomeric DNA in the various fractions was quantitated by ImageQuant analysis of PhosphorImager scans of spot blots hybridized with a mix of 5′-^32^P-(CCCTAA)_4_-3′ and 5′-^32^P-(TTAGGG)_4_-3′ radiolabelled oligonucleotides as described below (see Detection of telomeric and Alu repeat DNA). The fraction of telomeric DNA recovered was calculated as [bound÷(bound+unbound fraction)]; the unbound fraction was the total collected supernatant and wash fractions. For theoretical recovery calculations based on yields we reasoned that telomeres constitute about 0.026% of the total genome in our BJ-hTERT cell lines (based on the 17 kb average telomere length) and thus, a maximum yield of 26 ng pure telomeres is possible from 100 μg of genomic DNA. Actual yields were divided by 26 ng. The concentration of genomic DNA and recovered purified telomeres was quantitated using a Thermo Scientific NanoDrop 3300 fluorospectrometer which accurately measures concentrations in the picogram per μl range.

### Telomere restriction fragment analysis

Terminal restriction fragment analysis was performed using established protocols^26^,^56^. The exACTGene 24-kb Plus DNA molecular weight ladder (Thermo-Fisher Scientific), undigested genomic DNA (1 μg), digested genomic DNA (3 μg) or purified telomeres (1 ng) were separated by gel electrophoresis on a 0.6% agarose gel in 1 × TAE at 150 V for 30 min and 57 V for 23 h and 30 min. Gels were dried under vacuum at 80 °C for 2 h, stained with SYBR Green for 30 min at 42 °C, and imaged with a Typhoon fluorescent imager to visualize the molecular weight marker and undigested genomic DNA. Next, the gel was denatured in 1.5 M NaCl, 0.5 N NaOH, then neutralized in 1 M NaCl, 0.5 M Tris-HCl pH 7.0, and finally hybridized with a ^32^P-5′-(TTAGGG)_4_-3′ oligonucleotide probe as described below. Gels were subsequently washed and visualize via a Typhoon phosphorimager. Telomere length measurement was performed using ImageQuant and the Telorun program ( http://www4.utsouthwestern.edu/cellbio/shay-wright/research/sw_lab_methods.htm)^56^.

### UVC exposure of purified DNA *in vitro*

Purified genomic DNA from BJ-hTERT fibroblasts (125 μg) was diluted in 10 mM Tris-Cl, pH 8.0, 1 mM EDTA for a final concentration of 50 μg ml^−1^ and exposed 100 J m^−2^ UVC on parafilm. The DNA was then concentrated using Amicon Ultracel 30 k columns (Millipore), and 100 μg was used to purify telomeres as described above. For Fig. 4, restriction fragments of 1.6 kb containing 270 TTAGGG repeats (telomeric) or 1.5 kb of non-telomeric DNA (genomic) were obtained by digesting the pSXneo 270(T2AG3) plasmid (a generous gift from Dr Peter Lansdorp, BC Cancer Research Center) with BglII and XbaI or HindIII and PvuI restriction enzymes, respectively. The restriction fragments were purified using the GeneJET Gel extraction kit (Thermo Fisher). Fragments (25 μg ml^−1^) were preincubated in buffer (50 mM Hepes pH 7.5, 25 mM NaCl and 150 mM KCl) with or without 2 μM purified human TRF1 protein or BSA in a total volume of 10 μl for 45 min at room temperature. Reactions were then exposed to 100 J m^−2^ UVC on parafilm. The DNA was immunoblotted on N^+^ membranes for detection of CPDs and 6–4PPs and telomeric DNA (see below).

### Immunospot blot detection of DNA photoproducts

Immunospot blots of purified genomic, lambda and telomeric DNA were performed using the GE Manifold spot blot apparatus using an established method^57^. For each experiment nanogram amounts of telomeric DNA was loaded with the corresponding genomic DNA (loaded in duplicates) for each recovery time point. For 6–4PP detection purified telomeric fractions were combined from two independent exposure experiments (100 μg genomic DNA collected from each experiment for a total of 200 μg). Positively charged Hybond H^+^ membranes and Whatman filter papers (GE Healthcare Life Sciences) preincubated with 2 × SSC buffer were assembled onto the apparatus and heat-denatured (100 °C, 10 min) DNA samples were loaded on the membrane via vacuum blotting. Membranes were removed and placed DNA face-down on filter papers saturated with denaturation buffer (1.5 M NaCl, 0.5 N NaOH) followed by neutralization buffer (1 M NaCl, 0.5 M Tris-HCl pH 7.0). Membranes were then vacuum dried between filter papers at 80 °C for 2 h. Dried membranes were blocked for 1 h with 5% non-fat dry milk in 1 × PBS tween 20 (PBST) and incubated overnight with primary antibody against CPDs (1:5,000, clone KTM53 Kamiya Biomedical) or 6–4PPs (1:5,000, clone 64M-2 Cosmo Bio). The 64M-2 antibody recognizes 6–4PPs in every dipyrimidine sequence^58^; and according to the manufacturer. Since sequence specificity data were lacking for the CPD antibody, we confirmed the KTM53 clone recognizes CPDs in every dipyrimidine sequence by immunoblotting UVC irradiated (500 J m^−2^) 39-mer oligonucleotides containing a single dipyrimidine type. The CPD signal intensity matched the order of lesion frequency as determined previously by HPLC: TT>TC>CT>CC^59^ (Supplementary Fig. 8). The Membranes were washed with PBST and incubated for 1 h with secondary antibody (peroxidase-conjugated AffiniPure Goat Anti-Mouse IgG (H+L) from Jackson ImmunoResearch ( #115-035-146) used at a 1:5,000 dilution.). Amersham ECL Prime (GE Healthcare) was used to enhance the peroxidase activity on the membranes that were immediately exposed to X-ray films (Phoenix Research products). Antibody signal intensities were quantified by ImageJ software. Blots were subsequently hybridized with a mix of ^32^P-radiolabelled 5′-(CCCTAA)_4_-3′ and 5′-(TTAGGG)_4_-3′ probes for telomeric DNA, and then a ^32^P-labelled probe complementary to Alu repeat DNA (Table 1), as described below.

### Detection of telomeric and Alu repeat DNA

Specific radiolabelled oligonucleotides (Table 1) were hybridized to the respective dried gels or membranes. The telomeric probes 5′-(CCCTAA)_4_-3′ and 5′-(TTAGGG)_4_-3′ and the Alu probe were radiolabelled by incubating 10 pmoles of each with 30 μCi of ^32^P-ATP, and 40 units of Optikinase (Affymetrix) in 50 μl of 1 × Optikinase buffer for 1 h at 37 °C. The reactions were heat inactivated for 20 min at 65 °C and purified using the Illustra MicroSpin G-25 Columns (GE Healthcare). The membranes or dried gel were incubated for 30 min at 42 °C, in the hybridization buffer (5 × SSC, 5 × Denhardt solution, 10 mM Na_2_HPO_4_, 1 mM Na_2_P_2_O_7_)^56^, and then overnight at 42 °C with 10 ml of hybridization buffer containing 25 μl of the radiolabelled probe reaction. Hybridizations with the Alu probe were at 60 °C. The membranes were washed two times in 2 × SSC, 0.1% SDS, two times in 2 × SSC and two times in 0.2 × SSC before visualization with a Typhoon phosphorimager and quantification of the signal intensities with ImageQuant software.

### Extrachromosomal telomeric G-circle detection assay

The protocol for G-circle amplification was conducted as described^60^, with slight modification. Briefly, 3 ng of genomic DNA or captured isolated telomeres from U2OS cells was added to reactions (10 μl) containing 0.2 mg ml^−1^ BSA, 0.1% Tween, and 0.2 mM each dATP, dCTP, dTTP and 1 × polymerase ϕ29 buffer (NEB). Reactions were initiated by the adding 7.5 U ϕ29 DNA polymerase (NEB) and incubated at 30 °C for 8 h, followed by 65 °C for 20 min. Reaction products were blotted and ultraviolet crosslinked on a nylon membrane and probed by hybridizing a ^32^P-end labelled 5′-(TTAGGG)_4_-3′ oligonucleotide at 65 °C as described above, to detect the C-rich products generated from the G-rich circular DNA templates.

### Gel shift assays

Recombinant 6 × -histidine tagged human TRF1 protein was purified using a baculovirus insect cell expression system and an AKTA Explorer FPLC (GE Life Sciences)^61^. The baculovirus construct for TRF1 expression was provided by Dr Titia de Lange (Rockefeller University, NY). Sf9 cells expressing TRF1 were lysed in Buffer A [20 mM sodium phosphate pH 7.4, 500 mM NaCl] containing 40 mM imidazole, 1% NP-40 and a protease inhibitor cocktail (Roche Molecular Biochemicals) for 30 min at 4 °C. Subsequent buffers included protease inhibitors, 1 mM AEBSF and 5 mM β-mercaptoethanol. After sonication, the lysate was centrifuged and the supernatant was loaded onto a HisTrap FF column (GE Life Sciences) pre-equilibrated with Buffer A containing 40 mM imidazole, followed by 7.5 column volumes each of Buffer A containing 60 mM and 80 mM imidazole for washes, and 145 mM imidazole for elution. Pooled TRF1 containing fractions were dialysed against 20 mM Hepes pH 7.9, 100 mM KCl, 3 mM MgCl_2_, 1 mM dithiothreitol and 20% glycerol. Protein concentration was determined by Bradford Assay (BioRad) and purity was determined by SDS-PAGE and Coomassie Blue staining. Oligonucleotides (Table 1) for substrate preparation were purchased from Midland Certified Reagents Co. The CTRL or CPD oligonucleotides were 5′-end labelled with [γ-^32^P]- ATP and Optikinase enzyme, and annealed to oligonucleotide TLS in a 1:2 molar ratio in 50 mM LiCl by incubating at 95 °C for 5 min and then cooling to room temperature. Reactions (10 μl) were performed in 20 mM HEPES, pH 7.9, 150 mM KCl, 1 mM MgCl_2_, 0.1 mM EDTA, 0.5 mM DTT, 5% glycerol, 0.1% NP-40 and 5% β-casein with substrate and protein amounts as indicated in the figure legends. The reactions were incubated at 4 °C for 20 min, and mixed with 2 μl 0.25% bromophenol blue. Samples were electrophoresed on a 5% 29:1 (bisacrylamide:acrylamide) native gel at 4 °C and 140 V for about 2 h in 1 × TBE buffer and visualized with a Typhoon 9400 Phosphorimager. Bound and unbound substrates were quantitated using ImageQuant software, and the per cent bound was calculated as the radioactivity amount in the bound band divided by total radioactivity in the lane, after correcting for background in the no enzyme control.

### Statistics

A two-factor Analysis of Variance was used to determine whether the difference between the curves for telomeric and genomic repair was significant at 99% confidence level. The two predictor variables were ‘type of DNA’ (genomic or telomeric) and ‘time’ (h). For Fig. 4 a one-factor ANOVA was used to determine whether the differences between telomeric DNA+TRF1 versus telomeric DNA+BSA, genomic DNA+TRF1 versus genomic DNA+BSA, and telomeric DNA +TRF1 versus genomic DNA +TRF1 were significant at a 99% confidence level. The predictor variable was ‘type of DNA fragment and type of protein’. For Fig. 6 a two-factor ANOVA was used to determine whether the difference between the binding curves was significant at 99% confidence level. The two predictor variables were ‘type of substrate’ and ‘protein concentration’.

## Additional information

**How to cite this article:** Parikh, D. *et al.* Telomeres are partly shielded from ultraviolet-induced damage and proficient for nucleotide excision repair of photoproducts. *Nat. Commun.* 6:8214 doi: 10.1038/ncomms9214 (2015).

## Supplementary Material

Supplementary Information Supplementary Figures 1-8, Supplementary Table 1, Supplementary Methods and Supplementary References.

## Figures and Tables

**Table 1:** Oligonucleotides used in the study.

	**Sequence** ( 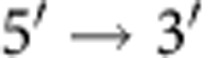 )
Ctrl	5′-GTGGATCCGTACTTAGGGTTAGGGTTAACACGAATTCGA-3′
CPD	5′-GTGGATCCGTACTTAGGGT<>TAGGGTTAACACGAATTCGA-3′
TPL	5′-TCGAATTCGTGTTAACCCTAACCCTAAGTACGGATCCAC-3′
Capture oligo (Telomere)	Bio-5′-ACTCC (CCCTAA)_3_-3′
Capture oligo (Scrambled)	Bio-5′-ACTCC(CATCAG)_3_-3′
^32^P- Telomere	5′-(TTAGGG)_4_-3′ and 5′-(CCCTAA)_4_-3′
^32^P–(Alu)_*n*_	5′-GGCCGGGCGCGGTGGCTCACGCCTGTAATCCCAGCACTTT-3′5′-GGGAGGCCGAGGCGGGCGGA-3′

T<>T denotes a CPD.
