# Supplementary material for: Telomeres are partly shielded from ultraviolet-induced damage and proficient for nucleotide excision repair of photoproducts
Source: Nat Commun. 2015 Sep 9;6:8214. doi: 10.1038/ncomms9214 (PMC4566151; doi:10.1038/ncomms9214)
Supplement: Supplementary Information — Supplementary Figures 1-8, Supplementary Table 1, Supplementary Methods and Supplementary References. [file ncomms9214-s1.pdf]

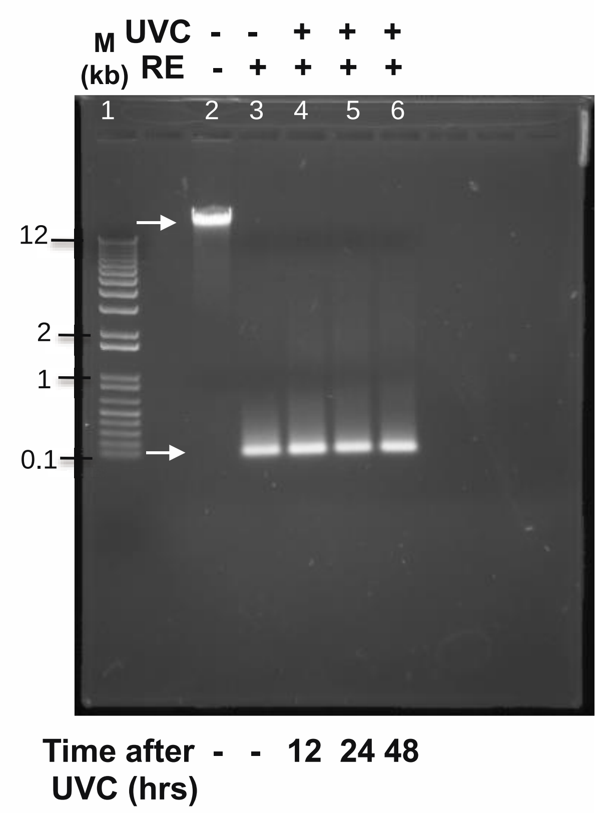

Supplementary Figure 1. Restriction enzyme digestion of genomic DNA. Genomic DNA isolated from untreated or 10 J m^{-2} UVC exposed BJ-hTERT cells and recovered at various time points (12 – 48 h) was digested overnight with a cocktail of four restriction enzymes as described in Methods. Aliquots of uncut or digested DNA (400 ng) were resolved on a 0.8% agarose gel at 110 V for 1 hour. Ethidium bromide staining reveals that the genomic DNA was digested to fragments < 1 kb in length.

a.

UVC J m $^{-2}$	lesion per 10 kb	CPDs per 10 kb (x 0.75)	6-4PPs per 10 kb (x 0.25)
0	0	0	0
5	1.7	1.2	0.42
10	3.3	2.5	0.62
20	6.5	4.8	1.6

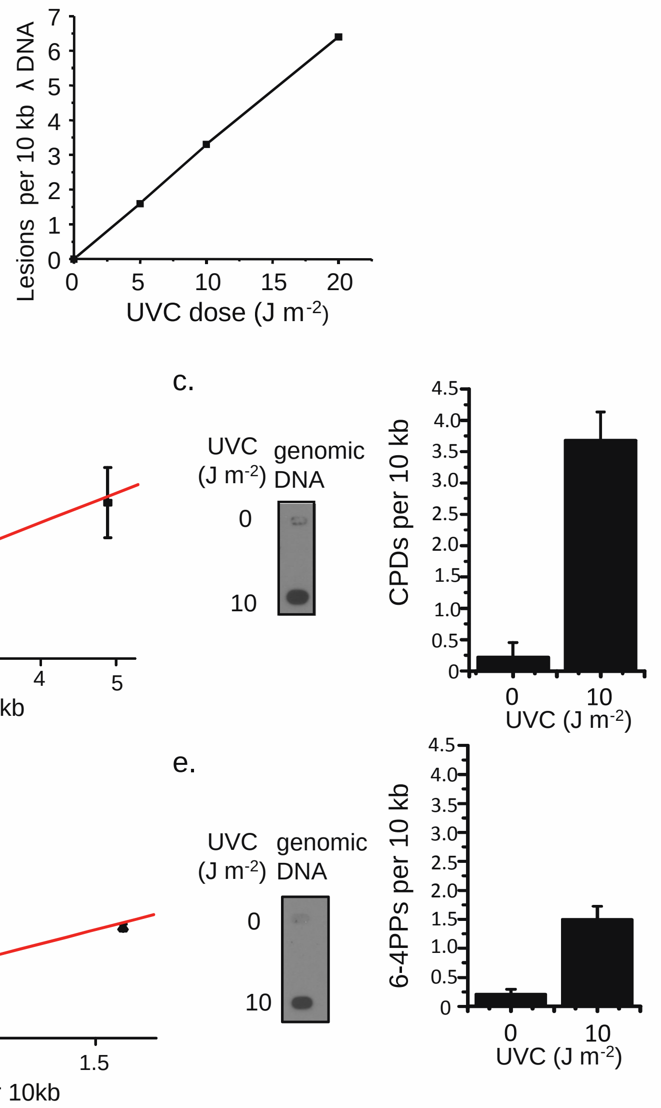

Supplementary Figure 2. **Quantification of CPDs and 6-4PPs in BJ-hTERT genomic DNA after cellular UVC exposure.** (**a**) Lambda DNA standards were prepared by exposing naked DNA to 0, 5, 10 or 20 J m $^{-2}$ UVC. Total UV photoproducts were measured by qPCR. The number of CPDs and 6-4PPs were deduced using the previously reported ratio of three CPDs for one 6-4PPs formed[1]. (**b**) Lambda DNA standards (3 ng) were loaded in triplicate on a membrane and immune-blotted for CPDs. Signal intensities (S.I.) were plotted as a function of the CPD number to generate a standard curve. (**c**) The CPD signal intensity was measured for BJ-hTERT genomic DNA from untreated or 10 J m $^{-2}$ UVC exposed cells (3ng) loaded on the same blot as the lamda DNA standards. The number of CPDs (right panel) was deduced from the lambda standard curve. (**d**) Lambda DNA standards (7 ng) were loaded in triplicate and immune-blotted for 6-4PPs. Signal intensities (S.I.) were plotted as a function of 6-4PP number to generate a standard curve. (**e**) The 6-4PP signal intensity was measured for BJ-hTERT genomic DNA from untreated or 10 J m $^{-2}$ UVC exposed cells (7ng) loaded on the same blot as the lamda DNA standards. The number of 6-4PPs (right panel) was deduced from the lambda standard curve. Values for lamda DNA standard curves represent the mean and error bars

represent the s.e.m. from three independent blots, and values from genomic DNA represent the mean and error bars represent the s.e.m. from three independent experiments.

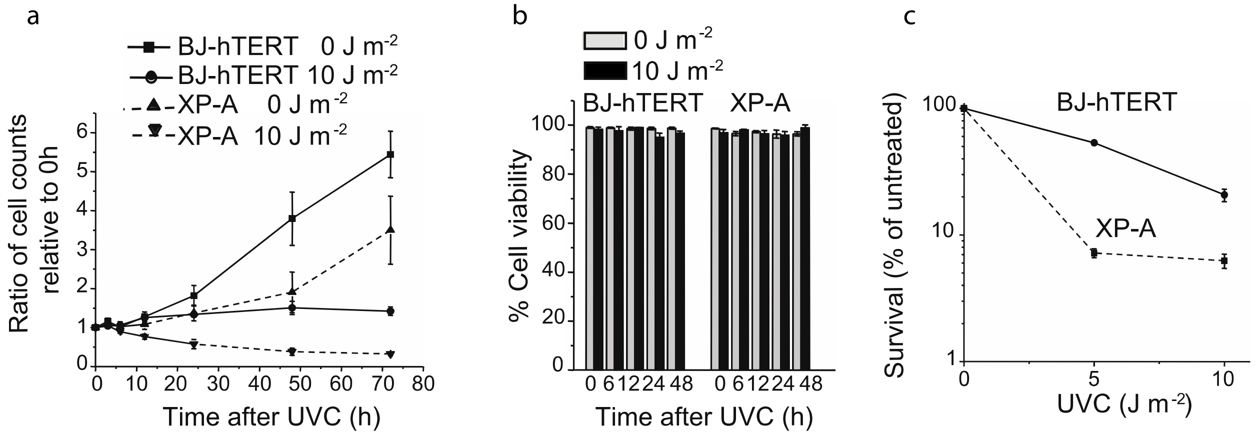

Supplementary Figure 3. UVC sensitivity and proliferation of BJ-hTERT and XP-A cells. (**a**) Cells were exposed to 10 J m^{-2} UVC, incubated in fresh media, and then counted after each recovery time point (0 - 72 h). Cell counts were normalized to the 0 h and plotted against recovery time. Values and error bars are from means and s.e.m. from three independent experiments. (**b**) Cells were exposed to 10 J m^{-2} UVC, incubated in fresh media, and then harvested after each recovery time point (0 - 48 h) by trypsinization after washing. Cells were counted manually on a hemocytometer for total cells and Trypan blue positive dead cells. Percent viability was calculated as described in Methods. Values represent the mean and s.e.m. from three independent experiments. (**c**) Cells were exposed to 0, 5, or 10 J m^{-2} UVC, recovered for 6h, sub-cultured and counted after 7 days of incubation. Survival was calculated as percent of untreated and plotted against UVC dose. Values and error bars are means and s.e.m. from three independent experiments.

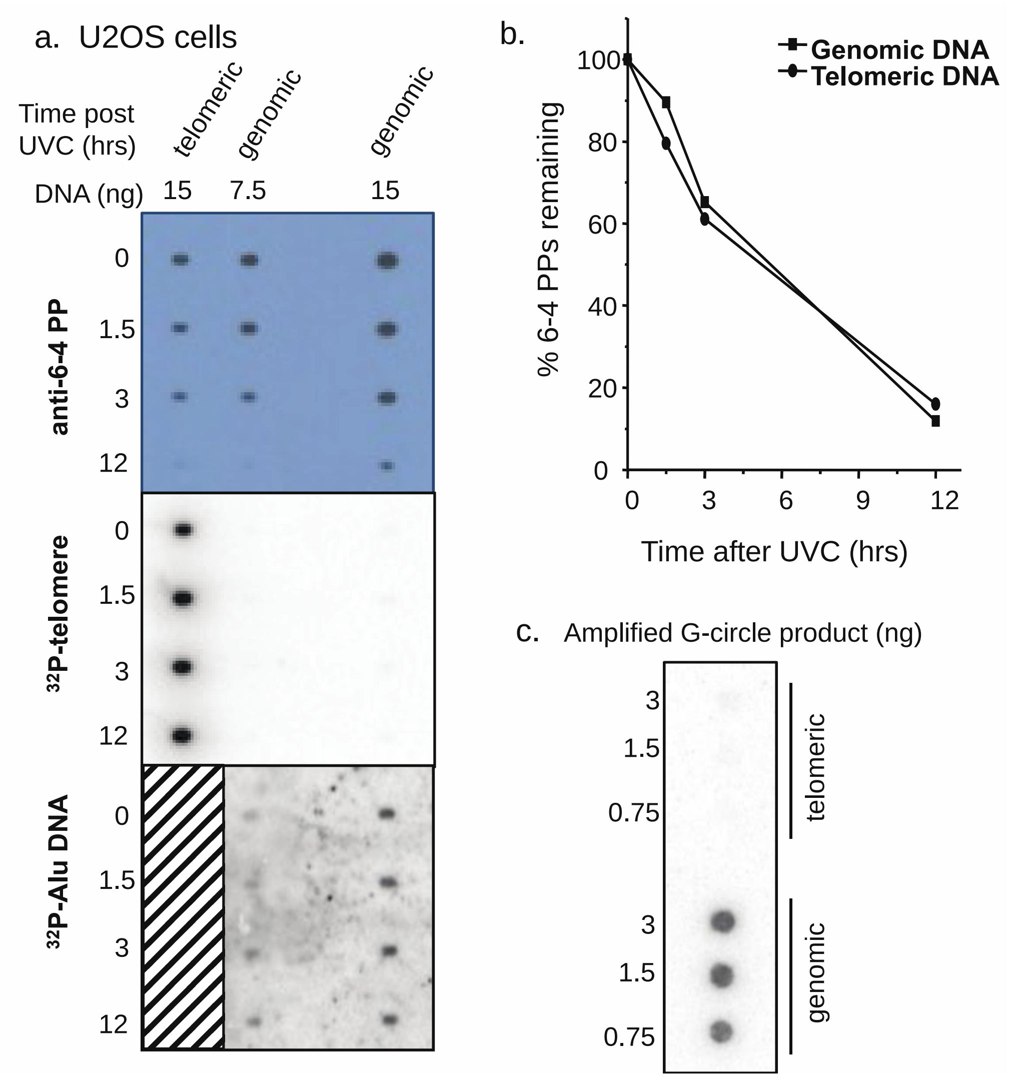

Supplementary Figure 4. Quantification of 6-4PP formation and removal in telomeric DNA from UVC exposed U2OS cells. (**a**) Cells were untreated or exposed to 10 J m^{-2} UVC and harvested at various repair times (0 - 12 h). Telomeres were isolated from purified genomic DNA (200 μg each time point) and loaded on a membrane (15 ng) (lane 1). Genomic DNA was loaded at 7.5 ng (lane 2) and 15 ng (lane 3). The blot was sequentially probed with a 6-4PP antibody, a radiolabeled telomere probe, and a radiolabeled Alu repeat probe. (**b**) The 6-4PP signal intensity was quantitated, normalized to 0 hour, and plotted against recovery time. (**c**) Amplification of ECTR G-circles by DNA polymerase ϕ29 rolling circle DNA replication for 8 hours with 3 ng genomic DNA or isolated telomeric DNA. Products (3, 1.5 and 0.75 ng) were blotted and detected with a radiolabeled probe.

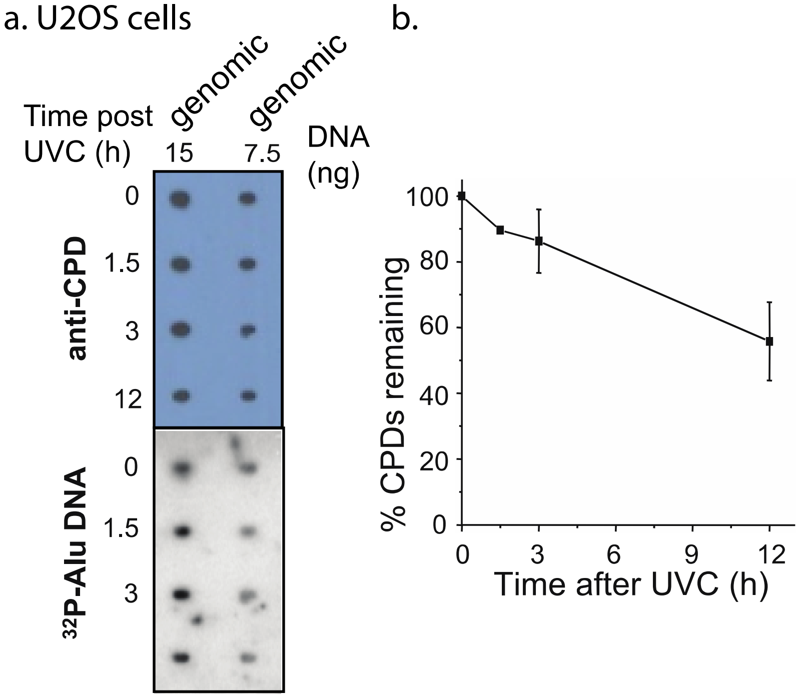

Supplementary Figure 5. Quantification of CPD formation and removal in genomic DNA from UVC exposed U2OS cells. Cells were exposed to 10 J m^{-2} UVC and harvested at various repair times. Genomic DNA was isolated and loaded (15 or 7.5 ng) on a membrane which was sequentially probed with CPD antibody and then a radiolabeled Alu repeat probe. The CPD signal intensity was quantitated, normalized to 0 hour, and plotted against recovery time. Values and error bars are means and s.e.m. from three independent experiments, except the 1.5 h time point which was from a single experiment.

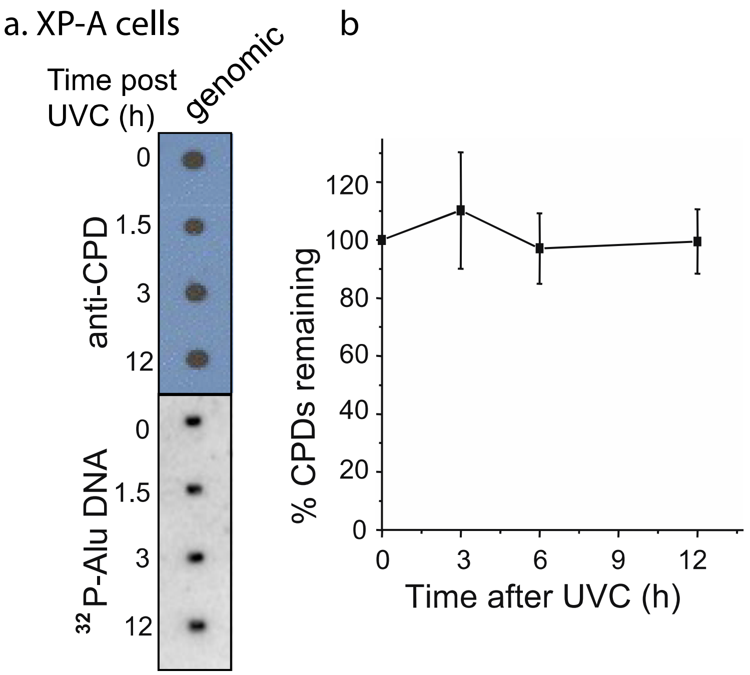

Supplementary Figure 6. Quantification of CPD formation and removal in genomic DNA from UVC exposed XP-A cells. Cells were exposed to 10 J m^{-2} UVC followed by harvesting at various repair times. Genomic DNA was loaded (10 ng) in duplicate on a membrane which was sequentially probed with CPD antibody and then a radiolabeled Alu repeat probe. The CPD signal intensity was quantitated, normalized to 0 hour, and plotted against recovery time. Values and error bars are means and s.e.m. from three independent experiments.

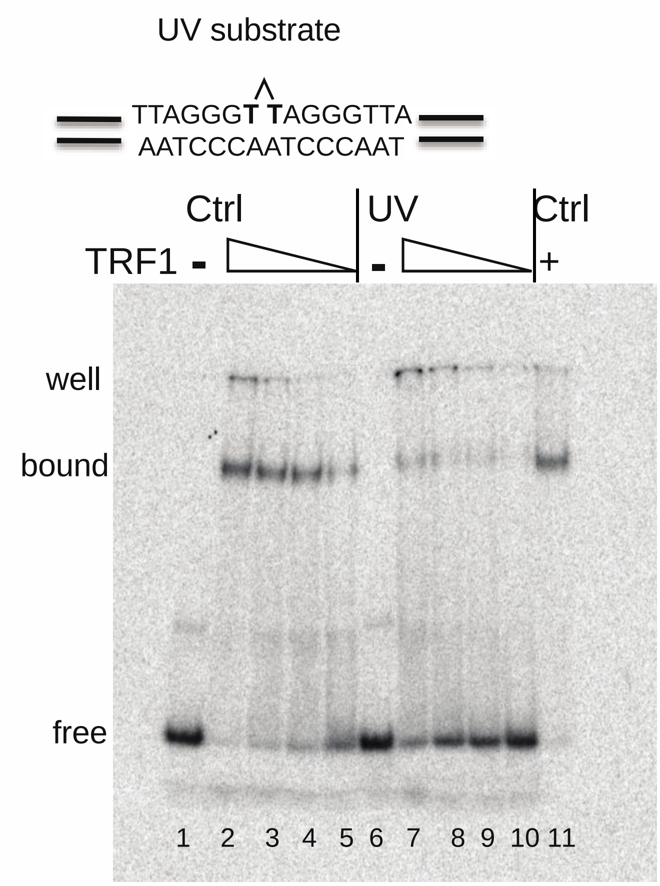

Supplementary Figure 7. Image of the uncropped gel from Figure 6. A cyclobutane pyrimidine dimer inhibits TRF1 binding to telomeric DNA. The TRF1 homo-dimer binding sequence and site specific CPD (^) is shown. Substrates (2.5 nM) consisting of annealed Ctrl/TPL (lanes 1-5, and 11) or UV/TPL (lanes 6-10) duplexes were incubated with decreasing TRF1 concentrations (200, 100, 50 or 25 nM) (lanes 2-5 and lanes 7-10) for 20 minutes in binding buffer, and reactions were run on a 5% acrylamide native gel. Lane 11 serves as a marker for bound TRF1, and includes the Ctrl substrate with 200 nM TRF1. Bound and unbound (free) substrate are indicated.

a.

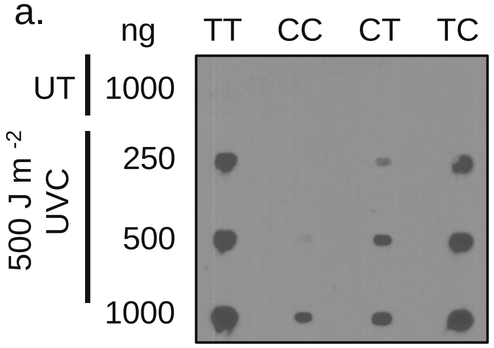

b. Oligonucleotide sequences 5’ to 3’

1. TT: AGAGTTAGATTAGAGTTAGATTAGAGTTAGAATTAGAAA

2. CC: TAGCCATGCCAGTGCCATATGCCATACCATGTACCATGT

3. CT: ATAGCTAGTGCTAGAGCTAGTGCTAGAGCTAGTGCTAGT

4. TC: ATAGTCAGAGTCATAGTCATAGTCAGAGTCATAGTCAGT

Supplemental Figure 8. The Kamiya KTM53 antibody recognizes CPDs in all dipyrimidine types. Four 39-mer oligonucleotides containing either six TTs, CTs, TCs, or CC and random sequence with no other dipyrimidines, were exposed to 500 J m^{-2} UVC at a 50 μg ml^{-1} concentration. (**a**) Untreated and irradiated DNA were loaded at various amounts and immuno-blotted with the Kamiya KTM53 anti-CPD antibody (1:5000 dilution). (**b**) Oligonucleotide sequences are shown 5’ to 3’, and dipyrimidine sites are underlined.

Supplementary Table 1. Percent of the telomeric and non-telomeric restriction fragment sequences containing dipyrimidine sites

Dipyrimidine 5' to 3'	$(TTAGGG)_{270}$ (1.6 kb)	Genomic (1.5 kb)
TT	16.7	13.9
TC	0	12.4
CT	16.7	13.8
CC	33.3	13.0
Total	66.7	53.0

The number of dipyirimidine sites of each type was counted for both strands of the $(TTAGGG)_{270}$ *Bgl*II and *Xba*I restriction fragment and the non-telomeric *Hind*II and *Pvu*I (genomic) restriction fragment of the pSXneo plasmid. This number was divided by the total number of nucleotides on both strands; 3240 for the telomeric fragment and 3010 for the genomic fragment.

Supplementary Methods

Quantitative PCR detection of DNA photoproducts. Quantitative PCR (QPCR) based quantification of DNA lesions on λ DNA (New England Biolabs) exposed *in vitro* to UVC irradiation was carried out based on a previously established method [2, 3]. The QPCR assay to quantify DNA lesions is based on the principle that any DNA lesion that greatly impedes or blocks progression of DNA polymerase will inhibit the extension step. To quantify UV-induced DNA lesions on λ DNA by QPCR, the forward and reverse primers used for amplifying the target from nucleotides 26890 to 39488 on the λ DNA template were: 5' CCA ACC ATC TGC TCG TAG GAA TGC 3', and 5' AGT TGG GTC CAC TTA TCG CGG AGT 3', respectively[3, 4]. The QPCR reactions were carried out using λ DNA template at 20 ng ml^{-1}. The cycling conditions were 75°C for 2 min, 94°C for 1 min, 94°C for 15 s and 66°C for 12 min (17 cycles), followed by 72°C for 10 min. Quantification of the λ DNA template and PCR products was performed using PicoGreen fluorescent DNA dye. The lesion frequency (lesions per strand, D) was calculated based on the "relative PCR amplification", which is the comparison between amplification of UV exposed samples with non-treated control. Quantification of the lesion frequency is based on the Poisson equation, which requires the assumption that DNA lesions are randomly distributed. The lesion frequency per DNA strand (average for both strands) is calculated as: *lesion frequency/amplified strand*: $D = -\ln (A_D/A_o)$, where A_D=amplification of damaged template, A_o=amplification of non-damaged template.

Supplementary References

1. Douki, T. & Cadet, J. Individual determination of the yield of the main UV-induced dimeric pyrimidine photoproducts in DNA suggests a high mutagenicity of CC photolesions. *Biochemistry* **40**, 2495-2501 (2001).
2. Ayala-Torres, S., Chen, Y., Svoboda, T., Rosenblatt, J. & Van Houten, B. Analysis of gene-specific DNA damage and repair using quantitative polymerase chain reaction. *Methods* **22**, 135-147 (2000).
3. Kad, N.M., Wang, H., Kennedy, G.G., Warshaw, D.M. & Van Houten, B. Collaborative dynamic DNA scanning by nucleotide excision repair proteins investigated by single-molecule imaging of quantum-dot-labeled proteins. *Mol Cell* **37**, 702-713 (2010).
4. Meyer, J.N. *et al.* Decline of nucleotide excision repair capacity in aging Caenorhabditis elegans. *Genome biology* **8**, R70 (2007).
